# Photobiomodulation 30 min or 6 h Prior to Cycling Does Not Alter Resting Blood Flow Velocity, Exercise-Induced Physiological Responses or Time to Exhaustion in Healthy Men

**DOI:** 10.3389/fphys.2020.607302

**Published:** 2021-01-15

**Authors:** Yago Medeiros Dutra, Gabriel Machado Claus, Elvis de Souza Malta, Daniela Moraes de Franco Seda, Anderson Saranz Zago, Eduardo Zapaterra Campos, Cleber Ferraresi, Alessandro Moura Zagatto

**Affiliations:** ^1^Post-Graduate Program in Movement Sciences, Laboratory of Physiology and Sport Performance (LAFIDE), Department of Physical Education, School of Sciences, São Paulo State University – UNESP, Bauru, Brazil; ^2^Post-Graduate Program in Movement Sciences, Department of Physical Education, School of Sciences, São Paulo State University – UNESP, Bauru, Brazil; ^3^Department of Physical Education, Federal University of Pernambuco, Recife, Brazil; ^4^Physical Therapy Department, Federal University of Sao Carlos (UFSCar), São Paulo, Brazil

**Keywords:** low-level laser therapy, exercise tests, ergogenic effects, regional blood flow, cardiorespiratory fitness

## Abstract

**Purpose:**

The aim of the current study was to investigate the effects of photobiomodulation therapy (PBMT) applied 30 min or 6 h prior to cycling on blood flow velocity and plasma nitrite concentrations at rest, time to exhaustion, cardiorespiratory responses, blood acid-base balance, and K^+^ and lactate concentrations during exercise.

**Methods:**

In a randomized, crossover design, 13 healthy untrained men randomly completed four cycling bouts until exhaustion at the severe-intensity domain (i.e., above respiratory compensation point). Thirty minutes or 6 h prior to the cycling trials, participants were treated with PBMT on the quadriceps, hamstrings, and gastrocnemius muscles of both limbs using a multi-diode array (11 cm × 30 cm with 264 diodes) at doses of 152 J or a sham irradiation (with device turned off, placebo). Blood samples were collected before and 30 min or 6 h after treatments to measure plasmatic nitrite concentrations. Doppler ultrasound exams of the femoral artery were also performed at the same time points. Cardiorespiratory responses, blood acid-base balance, and K^+^ and lactate concentrations were monitored during exercise sessions.

**Results:**

PBMT did not improve the time to exhaustion (*p* = 0.30). At rest, no differences were found in the peak systolic velocity (*p* = 0.97) or pulsatility index (*p* = 0.83) in the femoral artery, and in plasma nitrite concentrations (*p* = 0.47). During exercise, there were no differences for any cardiorespiratory response monitored (heart rate, *p* = 0.15; oxygen uptake, *p* = 0.15; pulmonary ventilation, *p* = 0.67; carbon dioxide output, *p* = 0.93; and respiratory exchange ratio, *p* = 0.32), any blood acid-base balance indicator (pH, *p* = 0.74; base excess, *p* = 0.33; bicarbonate concentration, *p* = 0.54), or K^+^ (*p* = 0.22) and lactate (*p* = 0.55) concentrations.

**Conclusions:**

PBMT at 152 J applied 30 min or 6 h before cycling at severe-intensity did not alter resting plasma nitrite and blood flow velocity in the femoral artery, exercise-induced physiological responses, or time to exhaustion in healthy untrained men.

## Introduction

Photobiomodulation therapy (PBMT) is a non-thermal electromagnetic radiation treatment that utilizes visible or invisible lights through laser or light-emitting diode sources (Anders et al., 2015). Although the use of PBMT has focused mainly on medical care, it has also been suggested as beneficial for exercise performance (Ferraresi et al., 2016). However, the findings supporting the effectiveness of PBMT in this scenario are not consistent. Several studies have demonstrated improved exercise performance after PBMT in open-chain isolated single-joint efforts (de Brito Vieira et al., 2014; Rossato et al., 2016), running (De Marchi et al., 2012; Dellagrana et al., 2018; Mezzaroba et al., 2018; Tomazoni et al., 2019), cycling (Lanferdini et al., 2018a), and sport-specific tests (Pinto et al., 2016). On the other hand, contradictory findings are also presented and highlighted year-by-year (Denis et al., 2013; da Silva Alves et al., 2014; Dos Reis et al., 2014; Malta et al., 2016; de Carvalho et al., 2020; Dutra et al., 2020).

The different settings of PBMT treatment parameters between studies, such as distinct irradiation power (i.e., amount of energy emitted by de device per second) and total energy delivered on the target tissues (energy [J] = power of irradiation [W] × duration of irradiation [s]) are generally used to explain the lack of agreement in the reported effects of PBMT on exercise performance (Ferraresi et al., 2016). However, *in vitro* assays and animal model studies (Ferraresi et al., 2015a,b) have suggested that the time between treatment and exercise may also be related to the inconsistent effects of PBMT in this scenario. These investigations demonstrated that the peak of PBMT efficacy in improving the transfer of hydrogen ions (H^+^) through the respiratory electron transport chain, adenosine triphosphate (ATP) synthesis (Ferraresi et al., 2015a), and physical performance (Ferraresi et al., 2015b) occurred 6 h after treatment. Therefore, an ideal time between treatment and exercise may play an important role in PBMT efficacy in improving human performance, in addition to an ideal configuration of treatment parameters. To date, the majority of investigations used PBMT up to 30 min before the exercise (Malta et al., 2016; Lanferdini et al., 2018a; de Carvalho et al., 2020; Dutra et al., 2020) and only one study has been conducted in humans addressing the suggested time-response effect of PBMT (Rossato et al., 2018). In this investigation, the authors observed that PBMT used 6 h or immediately before exercise reduced muscle fatigue induced by a knee extension-flexion exercise protocol in healthy men (Rossato et al., 2018). However, despite this promising result, findings on the effectiveness of PBMT in enhancing performance of whole body exercises such as cycling are inconsistent (Leal Junior et al., 2009; Teles et al., 2015; de Carvalho et al., 2020; Dutra et al., 2020). The current literature thus precludes definite conclusions about the PBMT time-response effect and its practical applications in whole-body exercises such as cycling, which renews the calls for research to assess this gap.

Likewise, despite the lack of agreement on treatment procedures, there is no clear understanding of how PBMT can enhance exercise performance. The mechanisms that usually explain its effects are essentially evidenced in *in vitro* and animal model studies and seem to be linked to improved cytochrome C oxidase (CCO) enzyme activity (Karu, 2010) and the release of nitric oxide from CCO and other stores such as heme proteins in the hemoglobin and myoglobin (Shiva and Gladwin, 2009). The increased activity of CCO leads to increased oxygen consumption (Wang et al., 2016; Linares et al., 2019) and rate of cellular ATP synthesis by the oxidative pathway (Ferraresi et al., 2015a,b). On the other hand, the increased bioavailability of nitric oxide may trigger signaling pathways to events such as vasodilation, which may improve tissue microcirculation (Maegawa et al., 2000) and tissue oxygenation (Wang et al., 2016; Linares et al., 2019). Based on these mechanisms, it is argued that PBMT may delay the onset of the fatigue process during exercise, increasing the time to exhaustion (Ferraresi et al., 2012). Theoretically, the improvements induced by PBMT in tissue oxygenation and rate of ATP synthesis by the oxidative pathway must be followed by a delayed increase in ATP synthesis by the non-oxidative systems and production of metabolic by-products such as lactate, inorganic phosphate (Pi), and hydrogen ions (H^+^). Moreover, the improvements induced by PBMT in nitric oxide bioavailability and tissue microcirculation would also lead to better clearance of these metabolic by-products from muscle. These physiological alterations would therefore lead to delayed disturbances caused by the metabolic by-products in muscle function, acid-base balance of muscle, and blood flow (e.g., decreased blood pH and bicarbonate concentration), and cardiorespiratory control (e.g., increased ventilation and carbon dioxide output due to the buffering of excess H^+^ released by muscle), which underpin the fatigue process during exercise (Boyas and Guével, 2011; Taylor et al., 2016; Hureau et al., 2018). A delay in these disturbances during exercise would therefore delay the onset of the fatigue process and increase the time until exhaustion (Ferraresi et al., 2012).

To date, some of the physiological alterations induced by PBMT that are linked to its ergogenic effects have been described in humans, such as increased tissue oxygenation (Wang et al., 2016; Linares et al., 2019). However, others have not been investigated such as vasodilation and nitric oxide bioavailability, which may be assessed by Doppler Ultrasound exam (Harris et al., 2018; Duarte-Mendes et al., 2020) and by the measure of plasma nitrite concentrations (an oxidative product of nitric oxide metabolism commonly used to assess its bioavailability) (Kelm, 1999), respectively. During exercise until exhaustion, the majority of studies focused on investigating PBMT effects on oxygen uptake $\left(\dot{\text{V}}\,{\text{O}}_{2}\right)$ and the reported findings are contradictory. Some studies have evidenced an increase in $\dot{\text{V}}\,{\text{O}}_{2}$ after PBMT (Lanferdini et al., 2018b; Mezzaroba et al., 2018; Tomazoni et al., 2019), whereas others have reported no beneficial effects (Malta et al., 2016; Dellagrana et al., 2018; de Carvalho et al., 2020). Other exercise-induced physiological responses such as increases in carbon dioxide output, ventilation, and blood lactate concentrations, as well as changes in blood acid-base balance, were also not affected by the PBMT (Dutra et al., 2020). However, all of these studies used PBMT a few minutes before exercise. The extent to which the time-response effect of PBMT influences physiological responses at rest and during exercises until exhaustion is currently unknown.

Thus, the purpose of the current study was to investigate the effects of PBMT applied 30 min or 6 h prior to cycling in comparison with a placebo (sham therapy) in time to exhaustion during severe-intensity exercise in healthy untrained males. Secondarily, we also addressed the effects of PBMT on resting and exercise physiological responses, which may be useful to explain its possible ergogenic effects. At rest, the plasma nitrite concentrations and the blood flow velocity in the femoral artery were assessed. During exercise, the cardiorespiratory responses and blood markers related to muscle fatigue were monitored. We hypothesized that PBMT would improve blood nitrite concentrations (i.e., due to suggested increase of PBMT in nitric oxide bioavailability) and decrease blood flow velocity at the femoral artery (i.e., due to suggested capacity of PBMT in induce vasodilation). During exercise, an increased time to exhaustion was expected after PBMT, followed by a lesser magnitude of cardiorespiratory and metabolic perturbations in analyses that were duration-matched. In addition, we also expected more expressive results with PBMT performed 6 h before exercise.

## Materials and Methods

### Participants

The required sample size was estimated using G^∗^power software (Faul et al., 2007). The input parameters used for *F* test family were: alpha = 0.05 and power = 0.80. Using the time until exhaustion during cycling at a maximal power output after placebo treatment (149 ± 23 s) and the one preceded by PBMT (171 ± 21 s) (Lanferdini et al., 2018a), the required sample size of 13 participants was estimated.

Fifteen healthy untrained men (mean ± standard deviation; age 24 ± 4 years; weight 74.1 ± 7.8 kg; height 175.8 ± 6.4 cm) with absence of vascular and metabolic disorders, musculoskeletal and joint injuries and recent (<6 months) and regular use of nutritional ergogenic supplementswere recruited to voluntarily participate in the current study. During the investigation, two participants were excluded due to regularly absent from the trials.

Before starting the study, participants were informed about the risks and benefits involved in the tests and then signed a consent form. All experimental procedures were approved by the São Paulo State University Research Committee (Protocol N°: 88446618.2.0000.5398) and conducted in accordance with the Declaration of Helsinki (1964).

### Experimental Design

**FIGURE 1:**
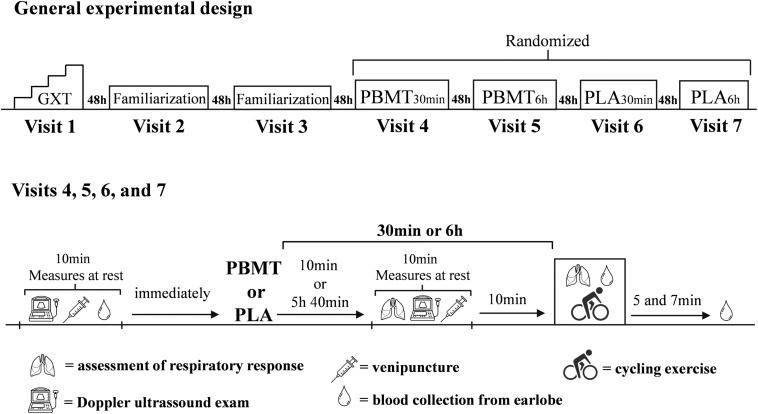
General experimental design of the study. GXT, graded exercise test until exhaustion; PBMT, photobiomodulation therapy; PLA, placebo.

The study was composed of seven visits to the laboratory, separated by 48 h. All methodological procedures were conducted in a double-blind, randomized, placebo-controlled design. Initially, participants completed a maximal graded exercise test with gas-exchange analysis to determine peak oxygen uptake $\left(\dot{\text{V}}\,{\text{O}}_{2\,p\,e\,a\,k}\right)$, maximal aerobic power, and respiratory compensation point power output. During the second and third visits, participants completed two bouts of cycling at severe-intensity (i.e., power output above respiratory compensation point) (Iannetta et al., 2019) until exhaustion as familiarizations. From the fourth to seven visits, participants underwent the same battery of cycling bouts and physiological assessments under four different conditions: PBMT applied 30 min before exercise (PBMT_30 min_); placebo (i.e., sham) intervention 30 min before exercise (PLA_30 min)_; PBMT applied 6 h before exercise (PBMT_6 h_); and placebo (i.e., sham) intervention 6 h before exercise (PLA_6 h_). Before treatments and 30 min (PBMT_30 min_ and PLA_30 min_ conditions) or 6 h after it (PBMT_6 h_ and PLA_6 h_ conditions), blood samples were collected by peripheral puncture of a forearm vein and a Doppler ultrasound exam of the femoral artery was performed. Respiratory responses during cycling trials were recorded andblood samples were collected from the earlobe before treatments and during exercise to measure blood acid-base balance, and lactate and potassium (K^+^) concentrations (Figure 1). All exercises were performed on an electromagnetic cycle ergometer (Lode-Excalibur Sport, Lode BV, Groningen, Netherlands) with a self-select cadence between 60–85 rpm, defined prior to beginning the experiments.

### Graded Exercise Test

The graded exercise test started at 75 W with increments of 25 W every 2 min until voluntary exhaustion, which was assumed as the inability to maintain the pre-set range cadence for 5 s (Miyagi et al., 2015). During the test, respiratory responses were recorded breath-by-breath using a stationary gas analyzer Quark CPET (Cosmed, Rome, Italy), previously calibrated according to the manufacturers’ instructions. The breath-by-breath data from each test were initially examined to exclude errant breaths caused by coughing, swallowing, and sighing, etc., and those values lying more than four standard deviations from the local mean were removed (Rossiter et al., 2002). The breath-by-breath data were subsequently interpolated every 1 s (Özyener et al., 2001) using the software OriginPro 8.0 (Origin Lab Corporation, Massachusetts, United States). Oxygen uptake $\left(\dot{\text{V}}\,{\text{O}}_{2}\right)$ was determined as the average of the final 30 s of each stage. The highest $\left(\dot{\text{V}}\,{\text{O}}_{2}\right)$ average attained during graded exercise test was assumed as $\left(\dot{\text{V}}\,{\text{O}}_{2\,p\,e\,a\,k}\right)$. The maximal aerobic power was assumed as the highest power reached during the graded exercise test. If a stage was not completed, maximal aerobic power was determined from (Eq. 1) (Kuipers et al., 1985). The ${\dot{\text{V}}}_{\text{E}}/\dot{\text{V}}\,{\text{CO}}_{2}$ *vs* power-output curve was divided into two-line regression and the respiratory compensation point was considered the intersection point (Binder et al., 2008).

(1) $$\begin{array}{c} \mathrm{Maximal}\,\mathrm{aerobic}\,\mathrm{power}=\mathrm{Final}\,\mathrm{stage}\,\left(\text{W}\right)+\\ \left[\left(\mathrm{duration}\,\mathrm{of}\,\mathrm{the}\,\mathrm{uncompleted}\,\mathrm{stage}\,\mathrm{in}\,\mathrm{seconds}/120\right)\cdot 25\right] \end{array}$$

### Cycling Exercise at Severe-Intensity

The exercise intensity was determined using (Eq. 2) as 60% of the difference between maximal aerobic power and power output at respiratory compensation point ([Δ60%], severe-intensity domain) (Dutra et al., 2020). This specific cycling until exhaustion protocol has been shown to elicit expressive metabolic responses and impairments in the neuromuscular system (Thomas et al., 2016; Dutra et al., 2020), and hence is useful for determining the impact of PBMT on performance and exercise-induced physiological changes. Before all exercises, participants performed a 5-min warm-up at 60% of maximal aerobic power, followed by four all-out sprints of 5 s. After a 4-min passive recovery, participants started the exercises. The time to exhaustion was recorded and expressed in seconds.

(2) $$\begin{array}{c} \mathrm{D60}\%=[(\mathrm{maximal}\mathrm{aerobic}\mathrm{power}-\\ \mathrm{respiratory}\mathrm{compensation}\mathrm{point}\mathrm{power}\mathrm{output})\times 0.6]\\ +\mathrm{respiratory}\,\mathrm{compensation}\,\mathrm{point}\,\mathrm{power}\,\mathrm{output} \end{array}$$

### Photobiomodulation Therapy and Placebo Interventions

**FIGURE 2:**
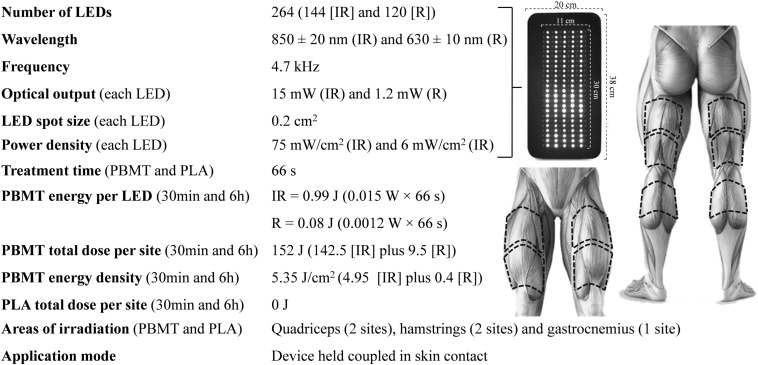
Parameters of PBMT device and treatment. IR, infrared; R, red; PBMT_30 min_, photobiomodulation applied 30 min before exercise; PLA_30 min_, placebo treatment applied 30 min before exercise; PBMT_6 h_, photobiomodulation applied 6 h before exercise; PLA_6 h_, placebo treatment applied 6 h before exercise.

The two PBMT and two PLA treatments were performed using a flexible multi-diode array *InLight* (Wellness Systems, United States). The multi-diode array (20 × 38 cm) was composed of 144 infraed diodes and 120 red diodes, each with an area of 0.2 cm^2^. The diodes were arranged uniformely in columns in the central area of the array, placed 1 cm apart. The central area of the array in which the diodes were arranged covered a rectanglular area of 11 × 30 cm (Figure 2). The treatments were performed on the quadriceps femoris (2 sites), hamstrings (2 sites), and gastrocnemius muscles (1 site), totalizing five irradiation site areas per lower limb. The placement of the multi-diode array over the target-muscles was performed identically to the description in Figure 2. The treatments lasted 66 s per site and the total treatment duration per condition was therefore 330 s. This duration enabled the irradition of 152 J of energy for each site. This amount of energy irradiated is in accordance with the recommendation of previous studies that suggest the deliver of 60 to 300 J for large muscle groups as effective to enhance exercise performance (Ferraresi et al., 2016; Vanin et al., 2018). PBMTs were applied with the device turned on, 30 min (PBMT_30 min_) or 6 h (PBMT_6 h_) before the exercise, or with the device turned off, 30 min (PLA_30 min_) or 6 h (PLA_6 h_) before the exercise. To ensure the double-blind design, all PBMT methods were conducted by a technician who did not perform any other procedures during the exercise sessions and data analysis. During all treatments, participants were blindfolded and wore headphones (listening to a standard song) to avoid the perception of any light or noise emitted by the device. The order of the PBMTs was determined using a simple randomization method (raffle), performed by a technician who also conducted all treatments (not involved in the assessment procedures and data analysis). The spectrum wavelength (850 nm for infrared diodes and 630 for red diodes) and power density (75 mW/cm^2^ for the infrared diodes and 6 mW/cm^2^ for red diodes) used are in accordance with the recommendations of previous studies (Zein et al., 2018; Hu et al., 2019).

### Doppler Ultrasound

Doppler ultrasound exam was performed in the femoral artery using a color Ultrasound Ge LOGIQ Book Xp/Xp (GE Healthcare, United States) with a linear transducer (8L-RS model) with a center frequency of ≈6.2 MHz. The width of the doppler window (i.e., gate) was the whole vessel. The probe was placed on the inguinal region and the common femoral artery was identified. After 5 min lying down, at rest, blood flow was recorded for 30 s continuous with the probe in the longitudinal plane, 1 cm above the vessel bifurcation (i.e., in superficial and deep femoral arteries), with an insonation angle of ≈60°. Ultrasound gel was applied between the transducer and the skin to ensure proper conduction of ultrasound signals. At least three measures in each exam were performed and the average between the three measures were calculated. After selecting a region of interest around the Doppler waveform, peak systolic velocity (the highest systolic velocity), and pulsatility index (Eq. 3), were automatically determined by the manufacturer’s software. If discrepancies were obtained between values (>8 cm/s for peak systolic velocity and >4 a.u. for pulsatility index), additional measurements were performed. The same examiner performed all ultrasonography procedures (not involved in the treatment procedures). Data are presented in absolute values and percent change after treatments.

(3) $$\begin{array}{c} \mathrm{Pulsatility}\mathrm{index}=(\mathrm{peak}\mathrm{systolic}\mathrm{velocity}-\mathrm{peak}\mathrm{diastolic}\\ \text{velocity})/\mathrm{mean}\mathrm{velocity} \end{array}$$

### Blood Collection and Analysis

For the measurement of plasma nitrite concentrations, blood samples were collected through peripheral puncture of a forearm vein into heparinized vacutainers and immediately centrifuged (4,000 rpm for 5 min) and stored at (−80°C) for posterior analysis. Nitrite, the first product of the reaction of nitric oxide with oxygen, commonly used to measure indirect nitric oxide levels (Bryan and Grisham, 2007), were measured in plasma using Griess reagent in which a chromophore with strong absorbance at 540 nm is formed by the reaction of nitrite with a mixture of naphtthylenediamine (0.1%) and sulfanilamide (1%), as previously described (Jacomini et al., 2017). Results were normalized by the total protein concentration (mg), determined by spectrophotometry using a random-access biochemical analyzer (A-15, BioSystmes, Spain).

Blood samples (25 μL) from the earlobe were collected through heparinized capillary tubes at rest, every 2 min of exercise, and 5 and 7 min after exercise. These collections were performed to, respectively, measure resting blood lactate concentration ([*L*a^–^]_rest_), the mathematical area under the curve (AUC) of [*L*a^–^] during exercise, and the peak of blood lactate concentration ([*L*a^–^]_peak_) after exercise. The AUC was calculated using OriginPro 8.0 software, plotting the results obtained (axis *Y*) *vs* the moments of the collection (axis *X*), deducting the rest concentration area. AUC was calculated according to the shortest exercise duration, ensuring that all conditions had the same number of points on the *Y* and *X* axes. Immediately after all collections, blood samples were deposited into microtubes containing 50 μL of sodium fluoride at 1%, and posteriorly analyzed in an electrochemical analyzer YSI 2,900 Stat Plus (Yellow Springs Instruments, Ohio, United States), being expressed in mmol/L.

For the assessment of blood acid-base balance and K^+^ concentrations, blood samples (60 μL) from the earlobe were collected through heparinized capillaries at rest and at a time correspondent to 80% of the time to exhaustion recorded during the second familiarization. Immediately after collections, samples were analyzed in a RAPIDLab 348EX gasometer (Siemens Healthcare Diagnostics, Camberley, United Kingdom) to measure blood pH, bicarbonate $\left({\text{HCO}}_{3}^{-}\right)$, base excess (BE) and K^+^ concentrations.

### Cardiorespiratory Responses During Severe-Intensity Cycling Exercise

During the cycling bouts, cardiorespiratory responses were recorded following the same procedures as in the graded exercise test. After the application of PBMT (before the cycling exercise), participants remained seated for 5 min to collect resting cardiorespiratory responses, calculated as the average of the data collected in the final 2 min. For analysis of cardiorespiratory responses, the breath-by-breath data from each test were initially examined to exclude errant breaths (i.e., values lying more than four standard deviations from the local mean caused by coughing, swallowing, sighing, etc.) (Rossiter et al., 2002) and subsequently interpolated every 1 s (Özyener et al., 2001) using the software OriginPro 8.0. The $\dot{\text{V}}\,{\text{O}}_{2},$ heart rate (HR), pulmonary ventilation $\left({\dot{\text{V}}}_{\text{E}}\right)$, carbon dioxide output $\left(\dot{\text{V}}\,{\text{CO}}_{2}\right),$ and respiratory exhange ratio (RER) were calculated for five time-windows during exercise (i.e., 20, 40, 60, 80, and 100%) as the average of the final 15 s of data of each moment. The times used to calculate these time-windows were similar between trials for each participant (i.e., isotime) by selecting the lower time to exhaustion attained during the four conditions. In addition, cardiorespiratory responses at exhaustion were also calculated as the average data of the final 15 s of exercise.

### Statistical Analysis

All data are presented as means and standard deviation (mean ± SD) with 95% confidence interval (CI95%). The impact of treatments on plasma nitrite concentrations, Doppler ultrasound exam outcomes, cardiorespiratory responses, blood acid-base markers, and blood K^+^ concentrations were tested with a two-way repeated measure analysis of variance (ANOVA). The impact of treatments on time to exhaustion and blood lactate concentrations were tested using a one-way repeated measures ANOVA. In all cases, the Mauchly’s test of sphericity was applied and the Greenhouse-Geisser Epsilon correction was used when the sphericity criteria were not met (Field, 2005). When necessary, the analyses were completed with SIDAK *post hoc*. The partial eta squared $\left(n_{p}^{2}\right)$ was reported and the threshold values were >0.001 (small), >0.06 (moderate), and >0.14 (large) (Cohen, 1988). The significance level of 5% was assumed in all cases. All statistical analyses were performed using the software package SPSS version 20.0 (IBM Corp., Armonk, NY, United States).

## Results

The $\dot{\text{V}}\,{\text{O}}_{2\,\text{peak}}$ reached in the graded exercise test was 37.82 ± 4.21 mL/kg/min (35.27 to 37.82 mL/kg/min) The maximal aerobic power and respiratory compensation point power output were 206 ± 32 W (186 to 225 W) and 167 ± 27 W (151 to 183 W), respectively. Based on maximal aerobic power and the respiratory compensation point power output, the exercise intensity corresponding to the cycling bout was 190 ± 29 W (172 to 208 W), which represented 92 ± 2.6% (91 to 94%) of maximal aerobic power. The time to exhaustion in familiarization one was 380 ± 71 s and in familiarization two was 396 ± 61 s (coefficient of variation equal to 8.1%).

### Plasma Nitrite Concentrations

There was no condition (*p* = 0.55, $n{}_{\text{p}}^{2}$ = 0.04), time (*p* = 0.90, $n{}_{\text{p}}^{2}$ < 0.01), or interaction (*p* = 0.48, $n{}_{\text{p}}^{2}$ = 0.07) effects for absolute values of nitrite blood concentrations. There were no differences between conditions in the percentage change values of plasma nitrite concentrations comparing the results obtained before and after treatments (*p* = 0.57, $n{}_{\text{p}}^{2}$ = 0.06) (Table 1).

**TABLE 1:** Blood nitrite concentration and Doppler Ultrasound exam outcomes of the femoral artery before and after treatments.

		PLA_30 min_	PBMT_30 min_	PLA_6 h_	PBMT_6 h_	ANOVA		
						Main Effect: Time	Main Effect: Condition	Interaction: Time × condition
Nitrite (nmol/mg of protein)	*Before*	0.38 ± 0.23 (0.23 to 0.53)	0.39 ± 0.31 (0.19 to 0.59)	0.33 ± 0.25 (0.17 to 0.49)	0.30 ± 0.18 (0.18 to 0.41)	*p* = 0.90 $n_{\text{p}}^{2}$ < 0.01	*p* = 0.55 $n_{\text{p}}^{2}$ = 0.04	*p* = 0.48 $n_{\text{p}}^{2}$ = 0.07
	*After*	0.34 ± 0.18 (0.22 to 0.46)	0.37 ± 0.33 (0.16 to 0.59)	0.33 ± 0.21 (0.20 to 0.47)	0.34 ± 0.20 (0.22 to 0.47)			
	Δ%	7.48 ± 58.75 (−29.84 to 44.81)	−7.64 ± 20.63 (−20.76 to 5.46)	28.25 ± 110.20 (−41.76 to 98.27)	32.54 ± 89.21 (−24.14 to 89.22)		*p* = 0.57 $n_{\text{p}}^{2}$ = 0.06	
***Doppler ultrasonography***								
Peak Systolic Velocity F.a (cm/s)	*Before*	65.05 ± 9.67 (59.20 to 70.90)	61.20 ± 11.31 (54.36 to 68.03)	74.86 ± 15.63 (65.41 to 84.31)	68.40 ± 18.85 (57.01 to 79.80)	*p* = 0.32 $n_{\text{p}}^{2}$ = 0.08	*p* = 0.02* $n_{\text{p}}^{2}$ = 0.25 (post hoc *P* ≥ 0.08)	*p* = 0.97 $n_{\text{p}}^{2}$ < 0.01
	*After*	60.33 ± 13.37 (52.25 to 68.42)	58.90 ± 17.81 (48.14 to 69.67)	70.89 ± 21.73 (57.75 to 84.02)	65.82 ± 18.06 (54.90 to 76.74)			
	Δ%	−7.20 ± 15.83 (−16.76 to 2.36)	−1.35 ± 32.18 (−20.80 to 18.09)	−2.50 ± 33.76 (−22.90 to 17.90)	−2.78 ± 41.36 (−22.21 to 27.78)		*p* = 0.82 $n_{\text{p}}^{2}$ = 0.09	
Pulsatility Index F.a (a.u.)	*Before*	11.08 ± 5.84 (7.55 to 14.61)	10.17 ± 4.83 (7.25 to 13.09)	9.53 ± 4.89 (6.57 to 12.49)	9.99 ± 3.23 (8.03 to 11.94)	*p* = 0.21 $n_{\text{p}}^{2}$ = 0.12	*p* = 0.85 $n_{\text{p}}^{2}$ = 0.02	*p* = 0.83 $n_{\text{p}}^{2}$ = 0.01
	*After*	12.10 ± 4.98 (9.08 to 15.11)	11.64 ± 5.31 (8.42 to 14.85)	11.03 ± 7.20 (6.67 to 15.38)	12.27 ± 7.69 (7.62 to 16.90)			
	Δ%	19.47 ± 43.02 (−6.52 to 45.46)	27.73 ± 72.87 (−16.66 to 71.41)	17.44 ± 50.37 (−13.00 to 47.88)	36.26 ± 84.07 (−14.54 to 87.07)		*p* = 0.82 $n_{\text{p}}^{2}$ = 0.02	

Values are expressed as mean ± SD (CI95%).

* = main condition effect; $n_{\text{p}}^{2}$, partial eta squared; F.a., femoral artery; PBMT_30 min_, photobiomodulation treatment 30 min before exercise; PLA_30 min_ = placebo treatment 30 min before exercise; PBMT_6 h_ = photobiomodulation treatment 6 h before exercise; PLA_6 h_ = placebo treatment 6 h before exercise *n* = 13.

### Doppler Ultrasound Exam of Femoral Artery

There was a condition effect on the absolute values of peak systolic velocity in the femoral artery (*p* = 0.02, $n_{\text{p}}^{2}$ = 0.25) but with no post hoc differences. There were no time (*p* = 0.32, $n_{\text{p}}^{2}$ = 0.08) or interaction (*p* = 0.97, $n_{\text{p}}^{2}$ < 0.01) effects on the absolute values of peak systolic velocity in the femoral artery. There were no condition (*p* = 0.85, $n_{\text{p}}^{2}$ = 0.02), time (*p* = 0.21, $n_{\text{p}}^{2}$ = 0.12), or interaction (*p* = 0.83, $n_{\text{p}}^{2}$ = 0.01) effects on the pulsatility index of the femoral artery. There were no differences between conditions in the percentage change values of both these parameters comparing the results obtained before and after treatments (Table 1).

### Performance and Exercise-Induced Physiological Responses

**FIGURE 3:**
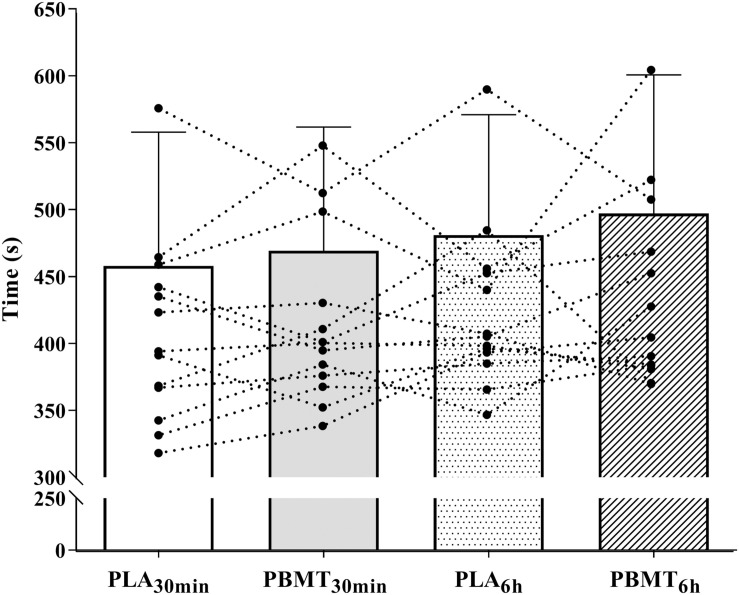
Time until exhaustion in severe-intensity cycling. PBMT_30 min_, photobiomodulation applied 30 min before exercise; PLA_30 min_, placebo treatment applied 30 min before exercise; PBMT_6 h_, photobiomodulation applied 6 h before exercise; PLA_6 h_, placebo treatment applied 6 h before exercise *n* = 13.

**FIGURE 4:**
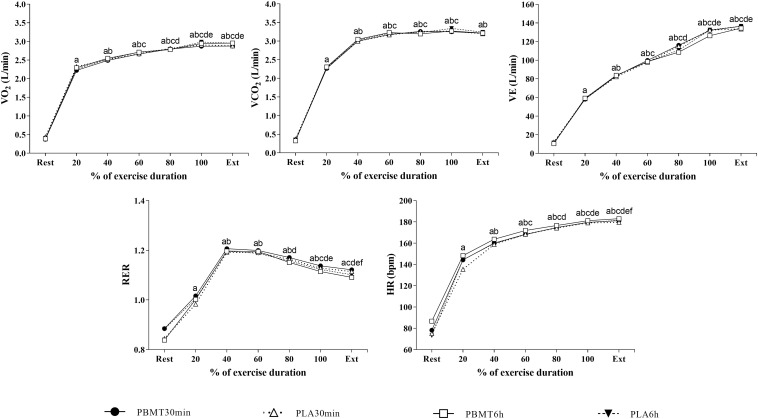
Cardiorespiratory responses measured during severe-intensity cycling until exhaustion. a = main time effect, significant difference from rest; b = main time effect, significant difference from 20% time-window; c = main time effect, significant difference from 40% time-window; d = main time effect, significant difference from 60% time-window; e = main time effect, significant difference from 80% time-window; f = main time effect, significant difference from 100% time-window; Ext, exhaustion; $\dot{\text{V}}\,{\text{O}}_{2}$, oxygen uptake; HR, heart rate; ${\dot{\text{V}}}_{\text{E}}$, pulmonary ventilation; $\dot{\text{V}}\,{\text{CO}}_{2}$, carbon dioxide output; RER, respiratory exhange ratio; PBMT_30 min_, photobiomodulation applied 30 min before exercise; PLA_30 min_, placebo treatment applied 30 min before exercise; PBMT_6 h_, photobiomodulation applied 6 h before exercise; PLA_6 h_, placebo treatment applied 6 h before exercise. *n* = 13. Error bars not included for clarity.

There were no differences in the time to exhaustion values (*p* = 0.30, $n_{\text{p}}^{2}$ = 0.09) (Figure 3). There was a time effect on HR (*p* < 0.01, $n_{\text{p}}^{2}$ = 0.98), $\dot{\text{V}}\,{\text{O}}_{2}$(*p*<$0.01,n_{\text{p}}^{2}=0.97)$, ${\dot{\text{V}}}_{\text{E}}$ (*p* < 0.01, $n_{\text{p}}^{2}$ = 0.96), $\dot{\text{V}}\,{\text{CO}}_{2}$ (*p* < 0.01, $n_{\text{p}}^{2}$ = 0.97) and RER (*p* < 0.01, $n_{\text{p}}^{2}$ = 0.87) (Figure 4). There were no condition or interaction effects on HR (*p* = 0.08, $n_{\text{p}}^{2}$ = 0.23; *p* = 0.15, $n_{\text{p}}^{2}$ = 0.20, respectively), $\dot{\text{V}}\,{\text{O}}_{2}$ (*p* = 0.22, $n_{\text{p}}^{2}$ = 0.11; *p* = 0.15, $n_{\text{p}}^{2}$ = 0.10, respectively), ${\dot{\text{V}}}_{\text{E}}$ (*p* = 0.69, $n_{\text{p}}^{2}$ = 0.03; *p* = 0.67, $n_{\text{p}}^{2}$ = 0.06, respectively), $\dot{\text{V}}\,{\text{CO}}_{2}$ (*p* = 0.93, $n_{\text{p}}^{2}$ = 0.01; *p* = 0.93, $n_{\text{p}}^{2}$ = 0.04, respectively), and RER (*p* = 0.30, $n_{\text{p}}^{2}$ = 0.09; *p* = 0.32, $n_{\text{p}}^{2}$ = 0.08, respectively).

There were no differences in [*L*a^–^]_rest_ (*p* = 0.24, $n_{\text{p}}^{2}$ = 0.10), AUC of [*L*a^–^] (*p* = 0.55, $n_{\text{p}}^{2}$ = 0.05), and [*L*a^–^]_peak_ (*p* = 0.95, $n_{\text{p}}^{2}$ < 0.01) (Table 2). A significant increase in blood K^+^ concentrations (*p* < 0.01, $n_{\text{p}}^{2}$ < 0.68), and a decrease in blood pH (*p* < 0.01, $n_{\text{p}}^{2}$ < 0.83), blood ${\text{HCO}}_{3}^{-}$ (*p* = 0.01, $n_{\text{p}}^{2}$ < 0.97), and BE (*p* = 0.01, $n_{\text{p}}^{2}$ < 0.98) concentrations were found comparing exercise and rest values. There were no condition or interaction effects for any blood marker measured. There were no differences between conditions in the percentage change values of blood K^+^ concentrations or blood acid-base balance indicators (Table 2).

**TABLE 2:** Blood metabolic indicators at rest and during severe-intensity cycling.

		PLA_30 min_	PBMT_30 min_	PLA_6 h_	PBMT_6 h_	ANOVA		
	
						Main Effect: Time	Main Effect: Condition	Interaction: Time × Condition
pH (a.u.)	*Rest*	7.46 ± 0.03 (7.44 to 7.48)	7.47 ± 0.02 (7.45 to 7.49)	7.46 ± 0.03 (7.44 to 7.48)	7.46 ± 0.03 (7.44 to 7.48)	*p <* 0.01 $n_{\text{p}}^{2}$ = 0.83	*p* = 0.74 $n_{\text{p}}^{2}$ = 0.04	*p* = 0.26 $n_{\text{p}}^{2}$ = 0.14
	*Exercise*	7.34 ± 0.06 (7.30 to 7.38)	7.34 ± 0.04 (7.31 to 7.37)	7.35 ± 0.04 (7.32 to 7.38)	7.35 ± 0.05 (7.32 to 7.39)			
	Δ%	−1.56 ± 0.97 (−2.18 to −0.94)	−1.77 ± 0.67 (−2.20 to −1.35)	−1.42 ± 0.81 (−1.94 to −0.90)	−1.47 ± 0.77 (−1.97 to −0.98)		*p* = 0.26 $n_{\text{p}}^{2}$ = 0.14	
${\text{HCO}}_{3}^{-}$ (mmol/L)	*Rest*	26.21 ± 2.20 (24.81 to 27.61)	26.85 ± 1.45 (25.92 to 27.77)	27.00 ± 1.69 (25.90 to 28.08)	26.74 ± 1.50 (25.78 to 27.69)	*p <* 0.01 $n_{\text{p}}^{2}$ = 0.97	*p* = 0.26 $n_{\text{p}}^{2}$ = 0.11	*p* = 0.55 $n_{\text{p}}^{2}$ = 0.06
	*Exercise*	19.10 ± 2.23 (17.68 to 20.51)	18.50 ± 1.52 (17.53 to 19.47)	19.21 ± 1.10 (18.51 to 19.91)	19.38 ± 1.28 (18.56 20.20)			
	Δ%	−26.17 ± 15.41 (−35.97 to −16.38)	−30.94 ± 6.01 (−34.77 to −27.21)	−28.63 ± 5.13 (−31.90 to −25.37)	−27.39 ± 5.14 (−30.66 to −24.12)		*p* = 0.46 $n_{\text{p}}^{2}$ = 0.06	
BE (mmol/L)	*Rest*	2.46 ± 1.58 (1.40 to 3.52)	2.73 ± 1.76 (1.55 to 3.92)	2.70 ± 1.86 (1.45 to 3.96)	2.75 ± 1.66 (1.63 to 3.87)	*p <* 0.01 $n_{\text{p}}^{2}$ = 0.98	*p* = 0.33 $n_{\text{p}}^{2}$ = 0.11	*p* = 0.53 $n_{\text{p}}^{2}$ = 0.07
	*Exercise*	−7.24 ± 2.11 (−8.66 to −5.82)	−7.50 ± 2.16 (−8.96 to −6.05)	−6.60 ± 1.37 (−7.53 to −5.68)	−6.44 ± 1.81 (−7.66 to −5.22)			
	Δ%	−906.75 ± 1415.22 (−1857.51 to 44.00)	−929.28 ± 1410.62 (−1876.95 to 18.39)	−1200.53 ± 2176.54 (−2662.76 to 261.68)	−450.81 ± 339.51 (−678.90 to −222.72)		*p* = 0.62 $n_{\text{p}}^{2}$ = 0.05	
K^+^ (mmol/L)	*Rest*	5.20 ± 0.43 (4.92 to 5.48)	5.12 ± 0.93 (4.53 to 5.72)	5.48 ± 0.73 (5.02 to 5.94)	5.21 ± 0.59 (4.83 to 5.59)	*p <* 0.01 $n_{\text{p}}^{2}$ = 0.68	*p* = 0.24 $n_{\text{p}}^{2}$ = 0.12	*p* = 0.22 $n_{\text{p}}^{2}$ = 0.11
	*Exercise*	5.67 ± 0.83 (5.14 to 6.20)	6.25 ± 0.63 (5.85 to 6.65)	5.97 ± 0.99 (5.34 to 6.60)	5.96 ± 0.67 (5.53 to 6.39)			
	Δ%	9.53 ± 16.82 (−1.15 to 20.22)	24.40 ± 16.70 (13.79 to 35.02)	10.26 ± 20.68 (−2.87 to 23.40)	15.46 ± 15.88 (5.36 to 25.55)		*p* = 0.15 $n_{\text{p}}^{2}$ = 0.15	
	[*L*a^–^]_rest_ (mmol/L)	0.71 ± 0.32 (0.51 to 0.91)	0.98 ± 0.43 (0.72 to 1.24)	0.97 ± 0.69 (0.55 to 1.39)	0.76 ± 0.27 (0.59 to 0.93)		*p* = 0.25 $n_{\text{p}}^{2}$ = 0.11	
	AUC of [*L*a^–^] (mmol/min)	22.40 ± 8.58 (17.22 to 27.61)	20.33 ± 7.81 (15.62 to 25.01)	19.84 ± 7.72 (15.10 to 24.50)	22.57 ± 11.64 (15.47 to 29.61)		*p* = 0.56 $n_{\text{p}}^{2}$ = 0.05	
	[*L*a^–^]_peak_ (mmol/L)	12.23 ± 1.85 (11.12 to 13.31)	12.39 ± 1.72 (11.30 to 13.39)	12.45 ± 1.95 (11.30 to 13.60)	12.33 ± 2.13 (11.04 to 13.6)		*p* = 0.95 $n_{\text{p}}^{2}$ = 0.01	

*Values are expressed as Mean ± SD (CI95%). $n_{\text{p}}^{2}$ = partial eta squared; ${\text{HCO}}_{3}^{-}$ = sodium bicarbonate; BE = base excess; K^+^ = potassium ion; [*L*a^–^]_rest_ = blood lactate concentration at rest; [*L*a^–^]_peak_ = peak of blood lactate concentration; AUC = area under curve; PBMT_30 min_ = photobiomodulation treatment 30 min before exercise; PLA_30 min_ = placebo treatment 30 min before exercise; PBMT_6 h_ = photobiomodulation treatmen 6 h before exercise; PLA_6 h_ = placebo treatment 6 h before exercise. *n* = 13.*

All raw data of blood nitrite concentrations, Doppler ultrasound results of the femoral artery, blood markers of exercise-induced metabolic responses, cardiorespiratory response, and time to exhaustion are shown in Supplementary Data Sheet 1.

## Discussion

The main findings of the current study were that, irrespective of whether applied 30 min or 6 h before exercise, PBMT did not change resting plasma nitrite concentration, blood flow velocity in the femoral artery, exercise-induced physiological responses during cycling at severe-intensity, or time to exhaustion. These findings refute our experimental hypotheses and highlight the necessity for careful interpretation of the previously reported PBMT ergogenic effects (Ferraresi et al., 2016).

Due to its short half-life, the bioavailability of nitric oxide in humans is commonly assessed by determining plasma nitrite concentrations (Kelm, 1999). Previous studies have demonstrated that resting plasma nitrite concentration can be modulated by acute interventions (Richards et al., 2018) and that its increase reflects changes in local nitric oxide production and blood flow (Lauer et al., 2013). Given that PBMT mechanisms are in part linked to improved nitric oxide bioavailability (Shiva and Gladwin, 2009; Buravlev et al., 2013), we expected increased plasma nitrite concentrations after PBMT treatments. However, no significant interactions were observed in this parameter. In line with the absence of resting plasma nitrite, blood flow velocity in the femoral artery also did not change. The blood flow velocity has been evidenced to be sensitive to changes in the vessel diameter (Hwang, 2017), with both the peak systolic velocity and pulsatility index decreasing when an increase in the vessel diameter occurs (Stebbings et al., 2013; Duarte-Mendes et al., 2020). Femoral artery flow accounts for ≈ 65% of leg flow (Zhang et al., 2008) and changes in leg muscle metabolism and microvasculature result in changes in femoral blood flow velocity (Dawson et al., 2013; Barton et al., 2018). According to *in* vitro and animal model studies, PBMT is able to induce vasodilation, which would change blood flow velocity and lead to lower values of both peak systolic velocity and pulsatility index in the femoral artery (Hwang, 2017). However, these changes were not observed in the present study. Unfortunately, we were unable to carry out assessments in microvasculature, or vessel diameter which could allow better determination of the impact of PBMT on local vasculature (Hildebrandt et al., 2017). Despite this, the novel results of the current study expand previous investigations (Larkin et al., 2012) and provide the foundation for future research.

According to the absence of changes in resting plasma nitrite concentrations and resting blood flow velocity in the femoral artery, the exercise-induced physiological responses monitored did not change with PBMT. During submaximal exercises above respiratory compensation point (severe-intensity) (Iannetta et al., 2019) performed until exhaustion, a progressive increase in the rate of ATP synthesis by the non-oxidative systems is observed (Burnley and Jones, 2007). This increase has been associated with the recruitment of the fast-twitch, less-efficient muscle fibers required for exercise maintenance (Burnley and Jones, 2007) and is followed by greater production of metabolic by-products such as lactate, Pi, and H^+^. In addition to the impairments in muscle contraction caused by the high-concentration of Pi into tissue (Allen et al., 2008), the excess of H^+^ induces changes in the acid-base balance of skeletal muscle and the bloodstream (e.g., decrease in blood pH and ${\text{HCO}}_{3}^{-}$ concentrations) and disturbances in cardiorespiratory control (e.g., increased ${\dot{\text{V}}}_{\text{E}}$ and $\dot{\text{V}}\,{\text{CO}}_{2}$ due to the buffering of excess H^+^ released by muscle). Collectively, these changes have been associated with the onset of the fatigue process during exercise (Boyas and Guével, 2011; Taylor et al., 2016; Hureau et al., 2018). Due to PBMT effects on tissue oxygenation (Wang et al., 2016; Linares et al., 2019) and rate of ATP synthesis by the oxidative pathway (Ferraresi et al., 2015b), we specifically expected greater $\dot{\text{V}}\,{\text{O}}_{2}$ together with a delayed increase in both ATP synthesis by the non-oxidative systems and production of metabolic by-products during exercise. Moreover, the expected improvements induced by PBMT in nitric oxide bioavailability (Shiva and Gladwin, 2009) would induce local vasodilation, improving the clearance of the metabolic by-products from lower limb muscles. These changes should be beneficial to exercise maintenance because they would delay the aforementioned disturbances and, thus, the onset of the fatigue process, increasing time to exhaustion. However, no significant differences were observed in any exercise-induced physiological response monitored.

The monitoring of physiological responses during exercise has been used to provide more insight into the PBMT ergogenic effects. The majority of previous studies have focused on investigating PBMT effects on $\dot{\text{V}}\,{\text{O}}_{2}$ at the end of graded-exercise tests. Some studies have evidenced greater $\dot{\text{V}}\,{\text{O}}_{2}$ after PBMT in these tests, whereas others have reported no effects on $\dot{\text{V}}\,{\text{O}}_{2}$ (Dellagrana et al., 2018; Miranda et al., 2016). During constant-load exercises, although improvements in $\dot{\text{V}}\,{\text{O}}_{2}$ on-kinetics have been evidenced after PBMT (Lanferdini et al., 2018b), no changes in $\dot{\text{V}}\,{\text{O}}_{2}$ and in other cardiorespiratory responses such as HR and carbon dioxide output were reported (Malta et al., 2016; Beltrame et al., 2018; Dutra et al., 2020). Given the magnitude of PBMT devices (e.g., single-diodes, cluster-diodes, and multi-diodes array) and treatment parameters such as spectrum wavelength (nanometers, nm), power of irradiation (milliwatts, mW), total energy delivered (joules, J), and number of applications, it is not surprising that results differ from one study to the next. In the present study, the spectrum wavelength (850 nm for infrared diodes and 630 for red diodes), power density (75 mW/cm^2^ for the infrared diodes and 6 mW/cm^2^ for red diodes), and total dose delivered (152 J for each muscle group) are in accordance with the recommendations of previous studies (Vanin et al., 2018; Zein et al., 2018; Hu et al., 2019). However, the sparse research addressing the effects of PBMT in human muscle tissue is inconclusive (Wang et al., 2016; Linares et al., 2019). Therefore, it is difficult to be certain if the recommended procedures carried out in the current study were sufficient to deliver an adequate amount of treatment to the active musculature. Thus, the lack of positive findings in the present study in exercise-induced physiological responses and time to exhaustion is intriguing but could be a consequence of inadequate treatment parameters. To date, the optimal PBMT treatment parameters are still unclear.

The effectiveness of PBMT to enhance performance has been suggested (Ferraresi et al., 2016; Vanin et al., 2018) but the supportive findings are not consistent (Teles et al., 2015; Malta et al., 2016; Lanferdini et al., 2018b; de Carvalho et al., 2020; Dutra et al., 2020). In addition, positive results come mainly from studies performed with single-joint exercises (Ferraresi et al., 2016; Vanin et al., 2018), which differ in many ways from sport-specific exercises (Sousa et al., 2017). For example, the total work performed by participants in a previous study during a knee-extensor fatiguing protocol was ∼ 4.2 kJ (Baroni et al., 2010). On the other hand, the participants of the current investigation performed ∼122 kJ of total work during the severe-intensity cycling exercise, with a mean cadence of ∼75 rotations per minute, power output ∼213 W, and duration of ∼458 s (PLA_30 min_ condition). Therefore, the PBMT dosages used to improve the performance of single-joint exercises may not be sufficient to improve the performance of sport-specific exercises such as cycling.

To date, the time-response effect of PBMT on physical performance in humans had been addressed by only one investigation (Rossato et al., 2018), evidencing that PBMT applied 6 h and immediately prior to exercises attenuated the loss of force of knee extensors after an isokinetic fatigue protocol compared with control, placebo, and PBMT applied immediately before exercise. These findings also demonstrated that an isolated treatment of PBMT 6 h prior to exercise did not elicit an ergogenic effect in humans, but suggested that the time-response aspect may be used to provide a summing effect of PBMT (6 h + immediately). This summing effect, in other words, could be signaling a need for more energy delivered on the target tissue for PBMT to promote ergogenic effects in some cases such as in whole-body exercises and in untrained individuals. The effects of PBMT on [*L*a^–^]_peak_ after exercise could partially support the assumption that the delivery of more energy is required in whole-body exercises, since PBMT was effective in decreasing [*L*a^–^]_peak_ only after single-joint exercises (Higashi et al., 2013); this effect was not observed in the current investigation or previous studies with running and cycling exercises (Malta et al., 2016; Pinto et al., 2016; Dutra et al., 2020). On the other hand, the need to deliver more energy to the muscles of untrained individuals is hypothesized based on *in vitro* studies, which evidenced that tissues with fewer mitochondria respond worse to PBMT (Zein et al., 2018). Because untrained individuals present lesser predominance of oxidative fibers, they need to receive a greater amount of energy in the muscles for PBMT to provide ergogenic effects. However, this assumption still needs to be investigated in humans.

Finally, we are aware that the current study has some limitations. The 2-min step duration used during the graded-exercise test may have overestimated the power output at RCP (Keir et al., 2016). Although a single graded-exercise test can be used to estimate both maximal and submaximal endurance performance indicators, the optimal stage and test duration for each measure seem to be different (Jamnick et al., 2018). Likewise, we were unable to conduct more accurate measures related to the suggested effects of PBMT, such as the assessment of microvasculature and vessel diameter in the lower limb muscles, as well as ATP production. These outcomes would allow us to better determine the effects of PBMT on human physiology and performance. In addition, we were unable to recruit more aerobically trained subjects. Although we invited trained individuals, the majority did not agree to participate due to the prolonged changes in their training routine. Considering the specific adaptations induced by endurance-training in muscle mitochondrial content and capillary density, trained individuals may respond better to PBMT (Zein et al., 2018). Nevertheless, these limitations do not affect the interpretations and conclusions of the current study. Collectively, the novel results reported expand previous investigations and provide a solid foundation for continued research that will lead to standards for PBMT usage. Additional studies with designs that vary usage parameters (e.g., time of treatment before exercise, total dose delivered), recruit more aerobically trained participants, and utilize sport-specific exercises will lead to clearer understanding of whether PBMT can be beneficial for muscle fatigue and increasing performance.

In conclusion, the current findings demonstrated that PBMT applied 30 min or 6 h before exercise was ineffective in improving resting plasma nitrite concentration and resting blood flow velocity at the femoral artery. Likewise, neither application moment of PBMT improved exercise-induced alterations in cardiorespiratory responses, blood metabolic markers, or time to exhaustion during cycling at severe-intensity performed by untrained men. Thus, the study did not support the use of PBMT as an ergogenic strategy for improving cycling performance in healthy men. Until a sufficient amount of research delivers a consensus regarding the effectiveness of PBMT and practice standards, the technique should be used with caution if acute changes in physical performance are desired.

## Data Availability Statement

The original contributions presented in the study are included in the article/Supplementary Material, further inquiries can be directed to the corresponding author/s.

## Ethics Statement

The studies involving human participants were reviewed and approved by São Paulo State University Research Committee. The patients/participants provided their written informed consent to participate in this study.

## Author Contributions

YD, CF, and AMZ designed the study. YD, GC, EM, ASZ, DMdS, and CF participated of the data acquisition. YD, EM, EC, and AMZ drafted the manuscript. All authors critically reviewed the manuscript and approved the final manuscript as submitted.

## Conflict of Interest

The authors declare that the research was conducted in the absence of any commercial or financial relationships that could be construed as a potential conflict of interest.

## Supplementary Material

Supplementary Data Sheet 1 Raw data of blood nitrite concentration, Doppler ultrasound exam results of femoral artery, markersof exercise-induced metabolic responses, cardiorespiratory responses and cycling performance.
